# Anti-Inflammatory Mechanism Prediction of Sinomenine Based on Network Pharmacology and Its Biological Activity Verification

**DOI:** 10.3390/biology14050543

**Published:** 2025-05-13

**Authors:** Da Song, Afsar Khan, Ming-Hong Dong, Chuan-Wen Lei, Ting-Ting Feng, Ying Zhou, Xin Wei

**Affiliations:** 1 School of Pharmacy, Guizhou University of Traditional Chinese Medicine, Guiyang 550025, China; s.ongda@163.com (D.S.); dmh99102@163.com (M.-H.D.); leichuanwen@yeah.net (C.-W.L.); fengtingting040@gzy.edu.cn (T.-T.F.); 2 Guizhou Key Laboratory of Modern Traditional Chinese Medicine Creation, Guiyang 550025, China; 3 Department of Chemistry, COMSATS University Islamabad, Abbottabad Campus, Abbottabad 22060, Pakistan; afsarhej@gmail.com

**Keywords:** sinomenine, network pharmacology, medicinal isoquinoline, mechanism and pathway, anti-inflammatory

## Abstract

Mechanism analyses of traditional Chinese medicine (TCM) components have always been a challenge in pharmaceutical research. As for the well-known medicinal isoquinoline from the plant *Sinomenium acutum*, the possible mechanism of the compound sinomenine for targeting anti-inflammatory activities is rarely involved and is still unclear. In this article, a network pharmacological strategy collected the common targets between major inflammation models and the compound sinomenine. The results suggested that JAK2, MMP9, PTGS2, JAK1, MPO, and JAK3 are the key targets between sinomenine and the main inflammatory indications. Then, bioactivity validation was used to further clarify the possible anti-inflammatory activity of sinomenine by ELISA analysis. At the tested concentration on LPS-induced RAW 264.7 cells, sinomenine significantly inhibited the expression of inflammatory factors NO, TNF-a, and IL-6, some of which were equivalent to the positive drug dexamethasone. Meanwhile, RT-qPCR experiments found that sinomenine clearly affected the expression of JAK/STAT pathway factors, including JAK1, STAT3, PTGS2, and IL-6. Molecular docking was further used to speculate on the binding mechanism of the compound sinomenine with key proteins in the JAK/STAT pathway. This study integrated network pharmacology, *in vitro* activity assessment, mechanism validation, and molecular docking to identify the key targets and potential pathway for sinomenine in anti-inflammatory treatment.

## Simple Summary

Mechanism analyses of medicinal natural products have always been a challenge in pharmaceutical research. The potential targets of isoquinoline sinomenine were predicted in disease treatment using network pharmacology. Based on these screened key targets, the pathways of action for sinomenine were enriched. Then, a cell model and PCR analysis were used to validate the predicted pathways and sinomenine’s anti-inflammatory activity. As for the results, sinomenine significantly inhibited the expression of inflammatory factors and clearly affected the level of JAK/STAT pathway factors. Finally, molecular docking was used for analyzing the possible binding action of sinomenine with key proteins in the JAK/STAT pathway. This work may share successful experiences for mechanism exploration with similar compounds.

## 1. Introduction

Inflammation is a widespread physiological response that occurs when the body is stimulated by pathogens or endogenous signals [1]. Excessive or uncontrolled inflammatory reactions may result in a variety of acute and/or chronic inflammatory damage, which further lead to pathological symptoms in the heart, pancreas, liver, kidney, lung, brain, intestinal tract, and so on [2]. The development of anti-inflammatory drugs with clear mechanisms and precise therapeutic effects has continuously been a hot topic in pharmaceutical research. It is noteworthy that due to the multi-target characteristics of drugs, the analysis of the mechanism of action for anti-inflammatory lead structures is receiving increasing attention from pharmaceutical scientists.

**Figure 1:**
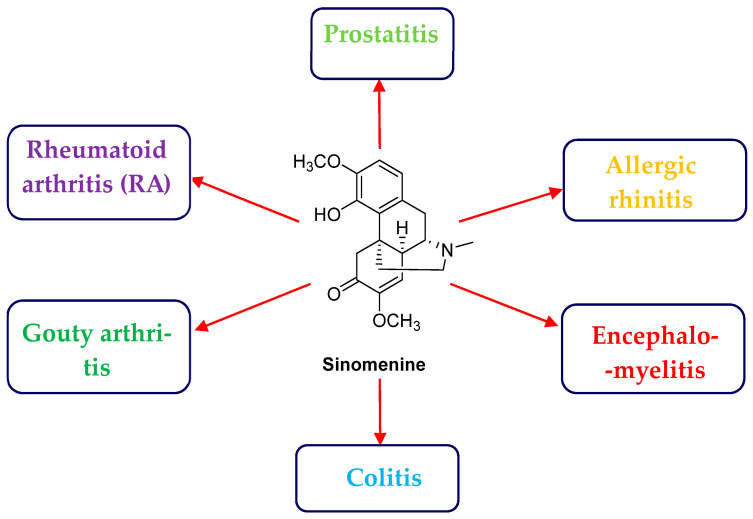
The structure of sinomenine and the selected common inflammatory diseases.

Based on several thousand years of clinical experience in traditional Chinese medicine (TCM), many natural products derived from TCM have been isolated and have been discovered to bear significant biological activity and controlling related diseases [3]. Among of them, the plant *Sinomenium acutum* from the family Menispermaceae is mainly distributed in East Asia, including China, Japan, South Korea, and so on. A large amount of anti-inflammatory alkaloids have been reported from this plant in the early stages of research. It is worth mentioning that the isoquinoline alkaloid sinomenine (SIN) (Figure 1) was also previously isolated and identified from the plant medicine *S. acutum* [4,5,6,7]. However, its possible common mechanism for targeting anti-inflammatory activities is rarely involved and is still unclear.

New findings from network information and artificial intelligence (AI) promoted the development of network pharmacology, which provided a feasible path for the mechanism analysis of lead compounds like sinomenine with a multi-targets feature [8,9]. Different from the previously reported works on sinomenine, network pharmacology was fully integrated with *in vitro* activity validation in this work to systematically analyze the common anti-inflammatory mechanism of SIN. Specifically, a network pharmacological strategy was used to individually link the common targets between the compound sinomenine and the selected major inflammation models, including rheumatoid arthritis (RA), gouty arthritis, colitis, encephalomyelitis, allergic rhinitis, and prostatitis (Figure 1). An intersection target Venn analysis further suggested that JAK2, MMP9, PTGS2, JAK1, MPO, and JAK3 are the key targets from the common targets of each inflammation model. Meanwhile, the GO and KEGG analysis indicated that the compound sinomenine may exert its effect through the JAK/STAT pathway [10]. Bioactivity validation finally confirmed its possible anti-inflammatory pathway by ELISA and PCR analysis. At the tested concentration on lipopolysaccharide (LPS)-induced RAW 264.7 cells, sinomenine significantly inhibited the expression of inflammatory factors NO, TNF-a, and IL-6. Additionally, sinomenine clearly suppressed the classic factors of the JAK-STAT pathway. The binding mechanism between sinomenine and the key targets of the JAK/STAT pathway was also investigated using molecular docking. The JAK/STAT3 pathway, as an important inflammatory pathway, involves physiological processes, such as immunity, cell division, and cell death. Overall, the key targets and potential pathway for sinomenine in anti-inflammatory treatment have been elucidated herein by integrating network pharmacology, *in vitro* activity assessment, mechanism validation, and molecular docking, which will provide and inspire new ideas for the mechanism analysis of chemical components in TCM.

## 2. Materials and Methods

### 2.1. Targets Collection for Disease and Sinomenine

The SDF (PubChem CID 5459308) file of structure and SMILES {CN1CC[C@@]23CC(=O)C(=C[C@@H]2[C@@H]1CC4=C3C(=C(C=C4)OC)O)OC} for sinomenine was obtained from Pubchem platform (https://pubchem.ncbi.nlm.nih.gov/, accessed on 10 May 2024, 8 November 2024) Then, SDF file of sinomenine was imported into the SwissTarget Prediction database (http://www.swisstargetprediction.ch/, accessed on 8 November 2024) and Similarity Ensemble Approach (SEA) database (https://sea.bkslab.org/, accessed on 28 April 2025) to obtain the targets for treating diseases, which were used for cross checking with target information from PubChem database. The targets related to rheumatoid arthritis (RA), gout, colitis, encephalitis, allergic rhinitis, and prostatitis were obtained using GeneCards database (https://www.genecards.org/, RA: 8 November 2024; Gout: 16 November 2024; Colitis: 8 November 2024; Encephalomyelitis: 8 November 2024; Allergic rhinitis: 8 November 2024; Prostatitis: 8 November 2024).

### 2.2. PPI Network Construction

The related targets of sinomenine, as well as the targets of the diseases (rheumatoid arthritis (RA), gout, colitis, encephalomyelitis, allergic rhinitis, and prostatitis), were imported to Wei Sheng Xin (https://www.bioinformatics.com.cn/, accessed on 8 November 2024; 11 November 2024; 12 November 2024; 28 November 2024; 29 November 2024; 5 December 2024) and CNS (https://cnsknowall.com/#/HomePage, accessed on 28 November 2024) to perform visual analyses and draw the Venn diagram, which was also used to obtain the core anti-inflammatory disease targets of sinomenine. The selected core target was placed on the STRING platform (https://cn.string-db.org/, accessed on 30 April 2025, 12 November 2024, 16 November 2024, 28 November 2024) to build the protein–protein interaction (PPI) network. The minimum required interaction score was set to medium confidence (0.400). Then, the “*tsv” format files of PPI network was imported into software Cytoscape 3.10.1 (https://cytoscape.org/, accessed on 15 March 2024) to perform network topology analysis on PPI results using the function of “Network Analyzer”.

### 2.3. GO Enrichment and KEGG Analysis

The DAVID platform (https://davidbioinformatics.nih.gov/summary.jsp, accessed on 28 November 2024) was used to perform biological enrichment analysis on the core target site. The results of top five GO terms in *p*-Value were visualized and analyzed though Wei Sheng Xin (https://www.bioinformatics.com.cn/). The Wei Sheng Xin (https://www.bioinformatics.com.cn/) platform was also used to draw the Sankey dot plot of KEGG analysis.

### 2.4. RAW264.7 Cell Culture

The RAW264.7 macrophages (Center for Excellence in Molecular Cell Science, CAS, Shanghai, China) were added to suitable T25 culture bottles. Then, DMEM medium containing 10% fetal bovine serum (VivaCell, c04001-500, Auckland, New Zealand) was added RAW264.7 to culture in a 37 °C, 5% CO_2_ cell culture incubator. Passages were conducted every 1–2 days, and RAW264.7 cells with good growth status and normal morphology were taken for further experiments. RAW264.7 cells in logarithmic growth phase were seeded into a 96 well plate with 1 × 10^4^ cells per well and cultured in a 37 °C, 5% CO_2_ cell culture incubator for 24 h. The old culture medium was then discarded and different concentrations (0, 25, and 50 μM) of sinomenine (Lot: PS012335 from Chengdu Push Biotechnology Co., Ltd., Chengdu, China) containing serum medium were added. After continuing to cultivate for 24 h, the old culture medium was discarded and then 100 μL of DMEM medium containing 10% CCK-8 (UElandy Cat: C6005M and Lot: 240905Q01-01 from Suzhou Uelandy Biotechnology Co., Ltd., Suzhou, China) was added to each well. The wells were incubated in a dark incubator for 1 h and the absorbance (OD) at 450 nm was measured using enzyme-linked immunosorbent assay reader (Thermo Scientific Spectrophotometer, Type 1530), which was used to calculate the cell survival rate (%). Cell survival rate (%) = (absorbance of drug well − average absorbance of blank well)/(average absorbance of control well − average absorbance of blank well) × 100%.

### 2.5. RAW264.7 Cell Viability Assay

RAW264.7 cells in logarithmic growth phase were seeded into 96 well plates with 1 × 10^4^ cells per well and cultured in a 37 °C, 5% CO_2_ cell culture incubator for 24 h. The old culture medium was then discarded, and the wells were randomly divided into blank control group, lipopolysaccharide (LPS) (Solarbio Cat: No. L8880 and Lot: No. 3230506004 from Beijing Solarbio Science & Technology Co., Ltd., Beijing, China) model group, LPS + sinomenine-treated (12.5, 25, and 50 μM) group, and LPS + dexamethasone (Lot: C15130166 from Shanghai Macklin Biochemical Co., Ltd., Shanghai, China) at 50 μM positive group. The blank control group was cultured with normal cells. The LPS model group had LPS added with a final concentration of 500 ng/mL. In the drug and positive groups, the corresponding concentrations of sinomenine and dexamethasone were added after LPS (500 ng/mL) induction for 24 h. The old culture medium was then discarded, and 100 μL of DMEM medium containing 10% CCK-8 was added to each well. The cells were incubated in a dark incubator for 1 h, and the absorbance (OD) was measured at 450 nm using an enzyme-linked immunosorbent assay reader to calculate the cell survival rate (%). Cell survival rate (%) = (absorbance of drug well − average absorbance of blank well)/(average absorbance of control well − average absorbance of blank well) × 100%.

### 2.6. Inflammatory Factor Inhibition Assay

RAW264.7 cells in logarithmic growth phase were seeded into 96 well plates with 1 × 10^4^ cells per well. They were randomly divided into blank control group, LPS model group, LPS + Sinomenine (12.5, 25, and 50 μM) group, and LPS + dexamethasone (50 μM) positive group, with 9 replicates in each group. The cells were cultured in a 37 °C, 5% CO_2_ cell culture incubator for 24 h. The old culture medium was discarded, and LPS with a final concentration of 500 ng/mL was added to the LPS model group. In the drug and positive groups, the corresponding concentrations of sinomenine and dexamethasone were added after LPS (500 ng/mL) induction for 24 h. Then take the supernatant at 3500 rpm for 10 min and use the nitric oxide detection kit (Beyotime Cat: S0021S and Lot: Z938241113 from Shanghai Beyotime Biotechnology Co., Ltd., Shanghai, China) to detect the amount of NO.

RAW264.7 cells were seeded into 6-well plates at 2 × 10^5^ cells per well, with blank control group, LPS model group, LPS + sinomenine 12.5, 25, 50 μM group, and LPS + dexamethasone 50 μM positive group. After culturing in a 37 °C, 5% CO_2_ cell incubator for 24 h, the old culture medium was discarded. LPS with a final concentration of 500 ng/mL was added to the LPS model group. In the drug and positive groups, the corresponding concentrations of sinomenine and dexamethasone were added after LPS (500 ng/mL) induction for 24 h. Take the supernatant at 3500 rpm for 10 min. The cytokine kit were used to detect IL-6 (Mouse IL-6 ELISA KIT Cat: # ZC-37988 from ZCIBIO Technology Co., Ltd., Shanghai, China) and TNF-α (Mouse TNF-α ELISA KIT Cat: # ZC-39024 from ZCIBIO Technology Co., Ltd., Shanghai, China).

### 2.7. RT-qPCR Assay

RAW264.7 cells were seeded into 6-well plates at 2 × 10^5^ cells per well, with blank control group, LPS model group, LPS + sinomenine (12.5, 25, and 50 μM) group, and LPS + dexamethasone (50 μM) positive group. After culturing in a 37 °C, 5% CO_2_ cell incubator for 24 h, the old culture medium was discarded. LPS with a final concentration of 500 ng/mL was added to the LPS model group. In the drug and positive groups, the corresponding concentrations of sinomenine and dexamethasone were added after LPS (500 ng/mL) induction for 24 h. After collecting the culture medium, cells were rinsed with pre-cooled PBS to remove residual culture medium. Then, cells were collected by blowing and placing in a centrifuge tube at 1000 rpm for 5 min. The PBS of collected cells was discarded, and such cells were stored in a −80 °C freezer for further work. Total RNA was extracted from cells using the tissue/cell RNA rapid extraction kit (SparkJade Cat: AC0202-B and Lot: WVJWL from Shandong Sparkjade Biotechnology Co., Ltd., Jinan, China). According to the instructions of the kit (SparkJade Cat: AG0305-B and Lot: WTBPW from Shandong Sparkjade Biotechnology Co., Ltd., Jinan, China), glyceraldehyde-3-phosphate dehydrogenase (GAPDH) was selected as the internal reference gene. Real-time fluorescence quantitative PCR (SparkJade Cat: AH0104-B and Lot: WTBTU from Shandong Sparkjade Biotechnology Co., Ltd., Jinan, China) was used to quantitatively analyze the relative mRNA expression levels of IL-6, JAK1, STAT3, and PTGS2 in each group of cells. The PCR primers used in this study were synthesized by Shanghai Sangon Biotech Co., Ltd., Shanghai, China and Shanghai Generay Biotech Co., Ltd., Shanghai, China. The relevant sequences are listed in Table 1.

### 2.8. Statistical Analysis

Statistical analysis was conducted on the experimental data using SPSS 26 software, and normality tests were performed. Independent sample *t*-test was used for comparison between two groups, and one-way analysis of variance was used for comparison between multiple groups. *p* < 0.05 was considered statistically significant.

### 2.9. Molecular Docking

The SDF file of the 3D conformer for sinomenine was retrieved from the PubChem database as the ligand file. The 3D pdb files of protein receptors files for PTGS2 (5F19), JAK1 (6N7A), STAT3 (6NJS), and IL-6 (7NXZ) were downloaded and obtained from PDB database (https://www.rcsb.org/, accessed on 12 October 2024, 18 November 2024, 25 February 2025). The Pymol 3.0.3 software was used to remove ligands from protein complexes and process monomer compound structure files into pdb format for future docking. The Auto Dock Vina software was used to dock protein receptors and ligands (*n* = 10) (Table S1). The binding energy level was used to predict the degree of binding between small molecule ligands and protein receptors. If the binding energy is less than −1.2 kcal/mol (−5 kJ/mol), it indicates that the target compound has certain binding activity with the compound, and the lower the binding energy, the better the docking effect. The docking results was visualized using Pymol 3.0.3 and Protein Ligand Interaction Profiler (PLIP) (https://plip-tool.biotec.tu-dresden.de/plip-web/plip/index, accessed on 20 November 2024, 25 February 2025). The Wei Sheng Xin platform was used to draw the heatmap of the molecular docking results.

## 3. Results

### 3.1. Disease and Sinomenine Targets Analysis

**Figure 2:**
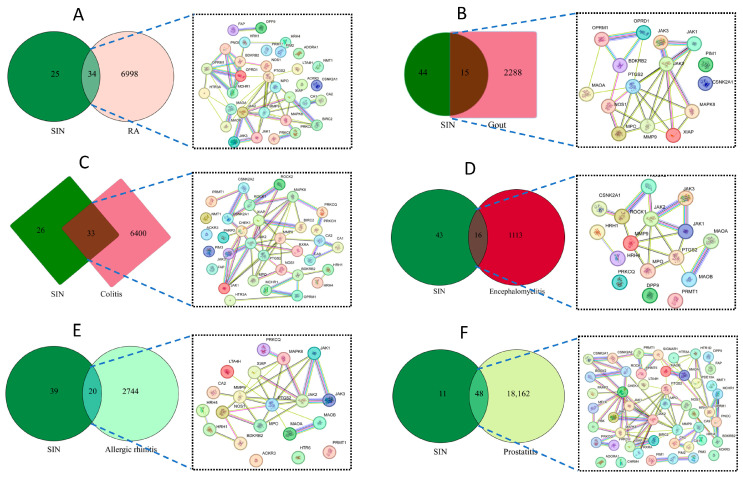
The common targets analysis between the selected main indications and sinomenine (SIN) (**A**) for rheumatoid arthritis (RA), (**B**) for gout, (**C**) for colitis, (**D**) for encephalomyelitis, (**E**) for allergic rhinitis, and (**F**) for prostatitis.

Rheumatoid arthritis (RA) is one of the most common autoimmune synovial diseases and is characterized by multiple, erosive, and chronic non-specific arthritis [11]. X-ray examinations of RA patients show osteoporosis and bone destruction. Previous studies have confirmed that the therapeutic effect of alkaloids on RA are mainly achieved by regulating immune cells, inflammatory cytokines, and the related signaling pathways [12,13]. The 59 targets of sinomenine and 7032 targets related to RA were obtained from the GeneCards database (https://www.genecards.org/). A total of 34 common targets between sinomenine and RA were collected (Figure 2A).

With the changes in modern lifestyles, gout has become a common metabolic disease. Abnormal purine metabolism caused an increase in blood uric acid, which may lead to the deposition of uric acid in joints, swelling, and pain, results in functional impairment [14]. Numerous works have suggested that alkaloids possess the potential to treat gouty arthritis [15,16,17]. A total of 15 common targets between the compound sinomenine and gout were collected (Figure 2B).

Colitis is a digestive system inflammation characterized by gastrointestinal adverse reactions as the main clinical manifestation [18]. The isoquinoline alkaloids have been reported to have significant therapeutic effects on colitis [19]. To clarify the possible key targets for sinomenine in colitis treatment, the 33 common targets of sinomenine and colitis were integrated into the STRING platform to establish a PPI network (Figure 2C).

In addition to the inflammatory models mentioned above, some studies have found that isoquinolines can also be used for the treatment of in vivo encephalomyelitis [20]. A total of 1129 targets relating to encephalomyelitis were obtained from the GeneCards database, and 16 common targets related to sinomenine and encephalomyelitis were collected from the intersection analysis (Figure 2D).

Allergic rhinitis is the common chronic inflammatory disease that affects human health. A reported study indicated that the natural product isoquinoline possessed good therapeutic activity in models of allergic rhinitis [21]. The 20 common targets of sinomenine and allergic rhinitis were collected from the GeneCards database and were visualized using Cytoscape 3.10.1 software (Figure 2E).

Additionally, the isoquinoline alkaloids have also been found to have certain applications and treatment potential in a prostatitis animal model [22]. The 48 common targets related to prostatitis and sinomenine were collected from the GeneCards database (Figure 2F).

More than half of the sinomenine targets were related to RA, colitis, and prostatitis. Additionally, more than a quarter of the sinomenine targets were related to gout, encephalomyelitis, and allergic rhinitis. Thus, sinomenine exhibited a strong correlation with the six indications mentioned above, which indicated that the selected diseases were representative for common pathways screening.

### 3.2. The PPI Network Construction, GO Enrichment, and KEGG Analysis

**Figure 3:**
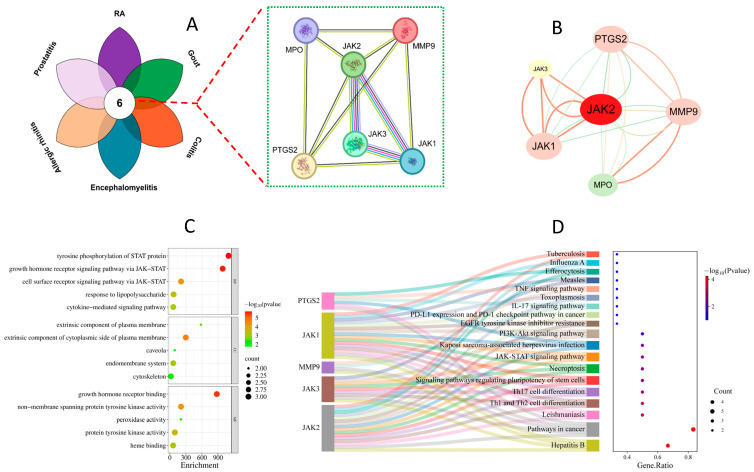
The PPI network, GO enrichment, and KEGG analysis for the core targets of sinomenine: (**A**) core target Venn diagram, (**B**) PPI network, (**C**) GO enrichment, and (**D**) KEGG analysis.

The targets crossover of sinomenine with rheumatoid arthritis (RA), gout, colitis, encephalomyelitis, allergic rhinitis, and prostatitis have resulted in six core anti-inflammatory disease targets (Figure 3A), which were used to construct its protein–protein interaction (PPI) network on the STRING platform. The PPI network involved 6 nodes and 22 edges. The small size of the PPI network is mainly due to the limited amount of selected core targets. The core targets were sorted in descending order of their degree value as JAK2, MMP9, PTGS2, JAK1, MPO, and JAK3 (Figure 3B) (Table S2). Among of these core targets, JAK2, JAK1, as well as JAK3, were important upstream factors in the JAK-STAT pathway [23], while PTGS2 (COX-2) was a downstream inflammatory factor in the JAK/STAT pathway [24,25].

Further GO functional enrichment have got 48 entries, including 33 for biological processes (BPs), 7 for cellular components (CCs), and 8 for molecular functions (MFs). The top five entries were shown in Figure 3C and suggested that the biological process (BP) of sinomenine mainly achieved through the JAK/STAT pathway [4]. Additionally, the KEGG analysis also indicated that significant enrichment was observed in the JAK/STAT signaling pathway (Figure 3D). Although other pathways were discovered in the KEGG analysis, the JAK/STAT pathway was still suspected as the predominant anti-inflammatory mechanism of SIN, which based on the fact that the core targets mostly belong to the JAK/STAT signaling pathway (Figure 3B).

### 3.3. Anti-Inflammatory Activity Evaluation of Sinomenine

**Figure 4:**
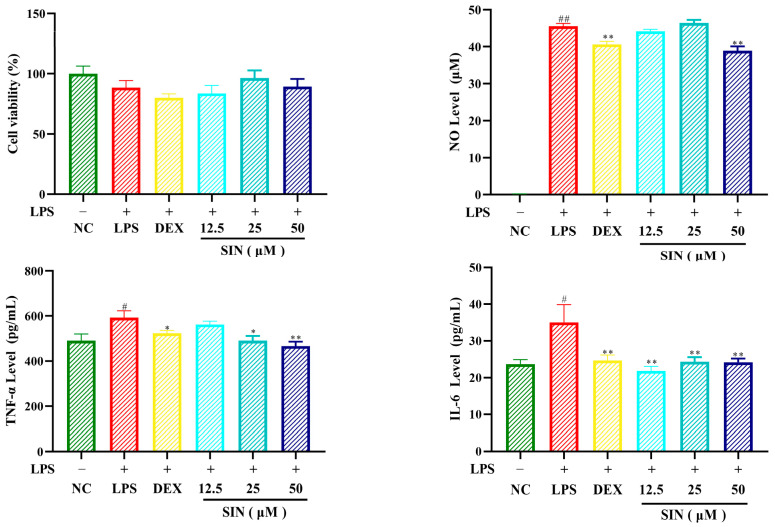
The inflammatory inhibition of sinomenine on the LPS-induced RAW264.7 cells; NC for negative control group, LPS for lipopolysaccharide induced model group, DEX for dexamethasone positive control group, SIN for sinomenine administration groups; compared to the NC group, # *p* < 0.05, ## *p* < 0.01; compared to the LPS group, * *p* < 0.05, ** *p* < 0.01.

To verify the anti-inflammatory activity of sinomenine (SIN), we established an inflammatory model on the LPS induced RAW264.7 cells. The factors NO, TNF-a, and IL-6 were tested at administration concentrations of 12.5, 25, and 50 uM, respectively [26]. As shown in Figure 4, sinomenine (SIN) have significantly inhibited the levels of inflammatory factors. It is worth mentioning that the inhibition of TNF-a by SIN was concentration dependent (Figure 4). In addition, the inhibitory activity of SIN on IL-6 was comparable to the positive drug dexamethasone (DEX) (Figure 4).

### 3.4. PCR Analysis and Pathway Confirmation

**Figure 5:**
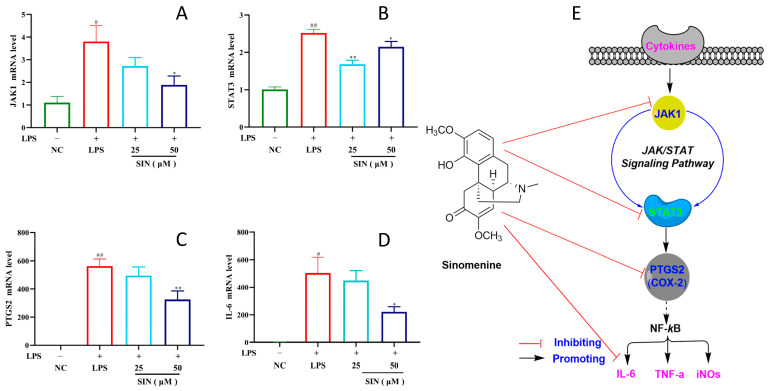
The PCR analysis of sinomenine related to the JAK-STAT pathway factors: (**A**) for JAK1, (**B**) for STAT3, (**C**) for PTGS2, (**D**) for IL-6, and (**E**) for the JAK-STAT pathway diagram; NC for negative control group, LPS for lipopolysaccharide induced model group, DEX for dexamethasone positive control group, SIN for sinomenine administration groups; compared to the NC group, # *p* < 0.05, ## *p* < 0.01; compared to the LPS group, * *p* < 0.05, ** *p* < 0.01.

According to the network pharmacology and molecular docking results, the PCR analysis was conducted on the important factors of the JAK/STAT signaling pathway. It was clearly detected that the expression of key upstream (JAK1) and/or downstream (STAT3, PTGS2, and IL-6) factors for JAK/STAT pathway were significantly inhibited under SIN dosage of 50 uM (Figure 5A–D) [27,28]. Such PCR experiments herein confirmed that the JAK/STAT pathway may be the main signaling pathway for the anti-inflammatory activity of SIN (Figure 5E), which may provide inspiration for future pharmaceutical applications research about SIN.

### 3.5. Molecular Docking

**Figure 6:**
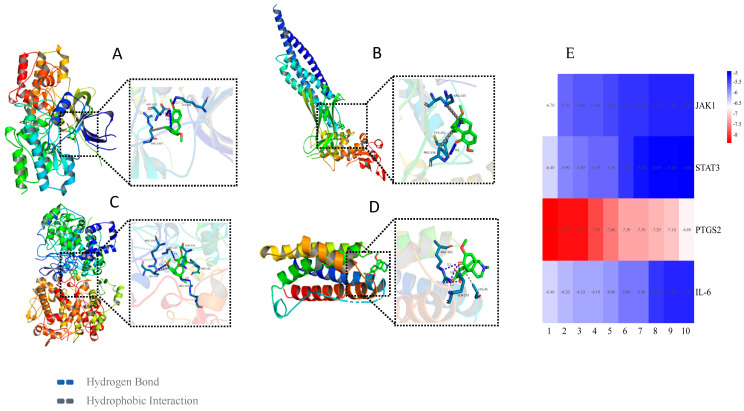
Molecular docking of key factor proteins of JAK-STAT pathway and compound sinomenine *(n* = 10): (**A**) for JAK1, (**B**) for STAT3, (**C**) for PTGS2, (**D**) for IL-6 proteins, and (**E**) for docking energy heatmap.

Based on the above analysis results of the network pharmacology and activity verification, the Auto Dock Vina v1.1.2 software was used to dock the key factor proteins (JAK1, PTGS2, STAT3, and IL-6) of the JAK/STAT pathway and compound sinomenine (Figure 6A–D). We predicted the degree of binding between the small molecule sinomenine and protein receptors based on the average binding energy level (*n* = 10). If the average binding energy was less than −1.2 kcal/mol (−5 kJ/mol), the docking substance showed certain binding activity. The lower the binding energy, the better the docking effect [29]. The results showed that the average binding energies between the targeted proteins and sinomenine were all below −5 kcal/mol (Table S1) (Figure 6E). In terms of docking actions, sinomenine was mainly linked with key factor proteins through hydrogen bonding and hydrophobic interactions. Specifically, sinomenine formed hydrogen bonds with the amino acid residues LYS908, CYS251, ARG376, and ARG103, which belong respectively to the proteins of JAK1, STAT3, PTGS2, and IL-6 (Figure 6A–D).

## 4. Discussion

The pharmacological effects of natural products from traditional Chinese medicine (TCM) have multi-target characteristics [30]. Therefore, mechanism analyses of active compounds have always been a difficult point and a challenge in pharmaceutical work. Network pharmacology is a discipline that has recently emerged based on the theory of systems biology, which analyzes the network of biological systems for multi-target drug molecule design and mechanism analyses [31]. With the development of artificial intelligence (AI) and big data in network pharmacology, its application on pharmaceutical mechanism research is becoming increasingly valued [32].

Sinomenine was previously isolated and identified as an isoquinoline alkaloid from the plant *S. acutum* [4]. In previous studies, a large amount of works have discovered anti-inflammatory clues related to sinomenine [33]. However, systematic analyses on its anti-inflammatory mechanism are still rare, especially for the common signaling pathway regulated by sinomenine in various reported inflammatory models. Therefore, to elucidate the possible anti-inflammatory mechanisms and pathways shared by sinomenine, network pharmacology was used to collect the common targets of the six main inflammatory diseases and sinomenine [10]. Further intersection analyses have resulted in the discovery of six potential key targets, which were responsible for the anti-inflammatory activity of sinomenine. Such key targets were used to construct its protein–protein interaction (PPI) network on the STRING platform. The core targets were sorted in descending order of their degree value as JAK2, MMP9, PTGS2, JAK1, MPO, and JAK3. Then, the GO enrichment and KEGG analysis were further conducted and suggested significant enrichment in the JAK/STAT signaling pathway. In view of this, we proposed the hypothesis that sinomenine might exert anti-inflammatory activity through the JAK/STAT pathway.

For these hypotheses and speculation, the anti-inflammatory activity of the compound sinomenine was tested and confirmed using *in vitro* anti-inflammatory evaluation on LPS-induced RAW 264.7 cells. Further PCR analyses revealed that the sinomenine significantly inhibited the key upstream and/or downstream factors of the JAK/STAT pathway [23,24,25,27,28], which supported and confirmed that the JAK/STAT pathway might be the main anti-inflammatory mechanism of sinomenine. Molecular docking provided obvious evidence of good affinity between sinomenine and regulatory factors in the JAK/STAT pathway [29]. As the key inflammatory factor of the JAK/STAT pathway, the factor IL-6 can promote JAK levels and then activate the JAK/STAT pathway. Although more evidence is needed, the current work suggested that SIN may reduce the expression of JAKs by inhibiting the production of IL-6, thereby blocking the activation of the JAK/STAT signaling pathway [4].

The generated data supported the potential application of SIN as a broad spectrum anti-inflammatory drug. It can be foreseen that our discovery will contribute to findings on the specific mechanisms of SIN aimed at the different indications above, and may particularly guide its in-depth preclinical and clinical research. In summary, these findings comprehensively utilized network pharmacology, molecular docking, *in vitro* activity evaluation, and mechanism validation to elucidate the possible anti-inflammatory mechanism of sinomenine, which may shed the new light for subsequent mechanism analyses of natural products with complex structures. The information generated by this article may open up future preclinical or clinical studies on diseases involving the possible mechanisms found and the factors studied.

## 5. Conclusions

In order to investigate the anti-inflammatory mechanism of sinomenine (SIN), network pharmacological strategy was used to discover the common targets between sinomenine and its reported indications, including rheumatoid arthritis (RA), gouty arthritis, colitis, encephalomyelitis, allergic rhinitis, and prostatitis. Further GO and KEGG analyses predicted that JAK/STAT might be the main anti-inflammatory signaling pathway for the compound sinomenine. Bioactivity validation *in vitro* finally suggested its anti-inflammatory activity on lipopolysaccharide (LPS)-induced RAW 264.7 cells. Additionally, PCR experiments further confirmed the mechanism of sinomenine on the JAK/STAT pathway. The binding mechanism between sinomenine and the key targets of the JAK/STAT pathway were also investigated using molecular docking. In summary, we have identified the possible mechanism of sinomenine by comprehensive network pharmacology, activity validation, and molecular docking, which will serve as useful experiences for mechanism studies on SIN in the treatment of rheumatoid arthritis (RA) and other similar diseases.

## Figures and Tables

**Table 1:** The PCR primer sequence.

Name	FORWARD	REVERSE
IL-6	CTTCTTGGGACTGATGCTGGTGAC	AGGTCTGTTGGGAGTGGTATCCTC
JAK1	CCCACTCCTTGATGCCAGTTCAC	CATGTCCGTCTTGCTCCGTCTTG
STAT3	GCAGAAGACACTGACTGATGAAGAG	AGACGGTCCAGGCAGATGTTG
PTGS2	GGTGCCTGGTCTGATGATGTATGC	GATGCTCCTGCTTGAGTATGTCG
GAPDH	CGATGCCCCCATGTTTGTGA	GAGCCCTTCCACAATGCCAA

## Data Availability

All data generated or analyzed during this study are included in this published article and its Supplementary Materials.

## Supplementary Materials

The following supporting information can be downloaded at: https://www.mdpi.com/article/10.3390/biology14050543/s1, Table S1 Molecular docking results of sinomenine with key factor proteins. Table S2 The degree values for each core node.

## Abbreviations

The following abbreviations are used in this manuscript:

AI	Artificial intelligence
BP	Biological process
CC	Cellular component
CCK-8	Cell Counting Kit-8
COX 2	Cyclooxygenase 2
DEX	Dexamethasone
ELISA	Enzyme-linked immunosorbent assay
GAPDH	Glyceraldehyde-3-phosphate dehydrogenase
GO	Gene ontology
IL-6	Interleukin-6
LPS	Lipopolysaccharide
JAK	Janus activated kinase
KEGG	Kyoto Encyclopedia of Genes and Genomes
MF	Molecular function
MMP 9	Matrix Metalloproteinases 9
MPO	Myeloperoxidase
PCR	Polymerase chain reaction
PLIP	Protein Ligand Interaction Profiler
PPI	Protein–protein interaction
PTGS 2	Prostaglandin-endoperoxide synthase 2
RA	Rheumatoid arthritis
RT-qPCR	Real-time reverse transcription polymerase chain reaction
SIN	Sinomenine
TCM	Traditional Chinese medicine
TNF-α	Tumor necrosis factor-α
