# Supplementary material for: Anti-Inflammatory Mechanism Prediction of Sinomenine Based on Network Pharmacology and Its Biological Activity Verification
Source: Biology (Basel). 2025 May 13;14(5):543. doi: 10.3390/biology14050543 (PMC12109165; doi:10.3390/biology14050543)
Supplement: Supplementary file 1 [file biology-14-00543-s001.zip › biology-3605650-supplementary.pdf]

Supporting Information

Anti-Inflammatory Mechanism Prediction of Sinomenine Based on Network Pharmacology and Its Biological Activity Verification

Da Song [1,2], Afsar Khan [3], Ming-Hong Dong [1], Chuan-Wen Lei [1,2], Ting-Ting Feng [1,2], Ying Zhou [1,2,*] and Xin Wei [1,2,*]

[1]School of Pharmacy, Guizhou University of Traditional Chinese Medicine, Guiyang 550025, China

[2]Guizhou Key Laboratory of Modern Traditional Chinese Medicine Creation, Guiyang 550025, China

[3]Department of Chemistry, COMSATS University Islamabad, Abbottabad Campus, Abbottabad 22060, Pakistan

*****Correspondence: yingzhou71@sina.com (Y. Zhou) and sfweixin@163.com (X. Wei).

Table S1 Molecular docking results of sinomenine with key factor proteins

Proteins	Molecular docking energy

JAK1 (6n7a)

mode	affinity (kcal/mol)	dist from best mode rmsd l.b.	rmsd u.b.
1	-6.7	0.000	0.000
2	-5.7	27.175	29.653
3	-5.6	43.088	45.270
4	-5.6	63.150	65.731
5	-5.5	59.555	61.719
6	-5.4	59.632	61.540
7	-5.4	64.092	66.125
8	-5.3	25.312	27.196
9	-5.3	21.059	23.613
10	-5.3	59.794	61.683

Writing output ... done.

STAT3(6NJS)

mode	affinity (kcal/mol)	dist from best mode rmsd l.b.	rmsd u.b.
1	-6.4	0.000	0.000
2	-5.9	18.926	21.395
3	-5.8	4.390	6.283
4	-5.7	47.383	50.304
5	-5.7	19.790	22.215
6	-5.4	14.271	17.298
7	-5.2	29.754	32.866
8	-5.1	19.367	21.542
9	-5.1	24.183	26.217
10	-5.0	23.368	25.298

Writing output ... done.

PTGS2 (5f19)

mode	affinity (kcal/mol)	dist from best mode rmsd l.b.	rmsd u.b.
1	-8.4	0.000	0.000
2	-8.2	14.714	16.564
3	-8.2	14.191	16.455
4	-7.9	14.823	17.075
5	-7.6	14.896	17.055
6	-7.3	3.041	5.718
7	-7.3	15.124	17.264
8	-7.2	2.441	5.209
9	-7.1	15.496	17.784
10	-6.8	15.163	17.414

Writing output ... done.

IL-6(7nxz)

mode	affinity (kcal/mol)	dist from best mode rmsd l.b.	rmsd u.b.
1	-6.4	0.000	0.000
2	-6.2	23.096	25.387
3	-6.1	11.929	14.682
4	-6.1	2.388	3.765
5	-6.0	20.105	22.568
6	-5.8	23.470	25.615
7	-5.8	20.607	22.866
8	-5.4	2.091	4.538
9	-5.3	12.460	15.286
10	-5.3	31.704	36.053

Writing output ... done.

Table S2 The degree values for each core node

Name	Degree	Radiality
JAK1	8	0.96
PTGS2	8	0.96
MMP9	8	0.96
JAK2	10	1
JAK3	4	0.8800000000000001
MPO	6	0.9199999999999999
